# Evaluating an intervention for healthcare professionals to facilitate family-centred conversations when an adult patient has a serious illness

**DOI:** 10.1186/s12909-026-09825-0

**Published:** 2026-07-17

**Authors:** Louise J. Dalton, Jeffrey R. Hanna, Rob J. Hallifax, Sophie Ratcliffe, Jack Arthey, Amalie Schumann, Elizabeth Rapa

**Affiliations:** 1 Department of Psychiatry, University of Oxford, Oxford, UK; 2 Institute of Nursing and Health Research, Ulster University, Belfast, UK; 3 South Eastern Health and Social Care Trust, Ulster Hospital, Dundonald, UK; 4 Oxford Centre for Respiratory Medicine, Oxford, UK; 5 National Institute for Health Research (NIHR) Oxford Biomedical Research Centre (BRC), Oxford, UK; 6 Faculty of English, University of Oxford, Oxford, UK

**Keywords:** Children, Serious illness, Communication, Healthcare professionals, Family-centred conversations, Intervention development, Evaluation, Parent

## Abstract

**Background:**

Many patients diagnosed with a serious illness have significant relationships with children (< 18 years old). While effective communication about an adult’s illness has psychological benefits for children, many families are uncertain about how to approach this topic. Patients frequently seek guidance from healthcare professionals, but clinicians often report a lack of knowledge, skills, and confidence to initiate such conversations. Consequently these discussions are often avoided.

A novel, brief intervention has been created to overcome these well-documented barriers. This multimethod study aimed to evaluate the acceptability, usefulness and effectiveness of an intervention for healthcare professionals.

**Methods:**

Volunteer sampling was used to identify and recruit healthcare professionals working with adult patients who have a serious illness. Participants completed an online survey regarding their current clinical practice, knowledge, skills and confidence about family-centred conversations before immediately accessing the intervention. Participants were invited to complete the same survey after accessing the intervention and give brief feedback about its usefulness. A subset of participants were contacted three months after their engagement with the intervention to participate in an online, individual interview. Non-parametric Wilcoxon signed-rank tests were conducted to evaluate differences between pre-post survey scores. Interviews were transcribed and analysed using reflexive thematic analysis.

**Results:**

Two hundred and fifty-five healthcare professionals completed the survey pre-post intervention. Quantitative responses indicated that the intervention had significantly increased participants’ knowledge, skills and confidence to initiate family-centred conversations. Over 90% reported that the intervention was relevant to their practice and 88% agreed they would recommend it to a colleague. Qualitative results (n=16) are discussed under three themes 1) perceptions of the intervention 2) perceived impact of the intervention on clinical practice 3) opportunities and challenges for family-centred conversations in routine practice. Participants commented favourably on the tone, format and content, with many describing a change in their practice.

**Conclusions:**

Healthcare professionals reported an increase in their knowledge, skills and confidence to initiate family-centred conversations after engaging with the intervention. Further evaluation is required to assess the sustainability of reported behaviour-change by healthcare professionals and its impact on patient and family outcomes.

**Supplementary Information:**

The online version contains supplementary material available at https://doi.org/10.1186/s12909-026-09825-0.

## Introduction

Many patients diagnosed with a serious illness such as cancer [1] or chronic respiratory disease [2] will have significant relationships with children (< 18 years old) in their role as a parent, grandparent, aunt or uncle. Despite evidence indicating the psychological benefits of effective communication with children about a parent’s illness [3], many families remain uncertain about whether to tell children about a health condition [3, 4]. Patients consistently describe wanting guidance from their healthcare team about talking to children [3–5]. However, healthcare professionals (HCPs) often feel ill-equipped to raise this topic in routine clinical care [6–10]. HCPs report insufficient knowledge and skills to advise patients about talking to children [11–15] as well as uncertainty about whether patients would welcome such a discussion [11]. Alongside this, many HCPs are unsure if this aspect of supportive care constitutes part of their role [6, 16, 17]. Consequently, these conversations are often avoided [11, 12], leaving families without support about talking to children [5].

Research has highlighted HCPs’ desire for training to develop their knowledge, confidence and skills around family-centred conversations [11–13, 15, 18]. There are a number of communication skills courses using different learning formats such as lectures, videos, structured communication frameworks and role play [18–22] for which evaluation data has been published. Some provide general communication skills [21], while others focus on skills for HCPs working in oncology or end of life services [23–25]. A systematic review only identified two educational interventions for HCPs which included specific content related to communication with children. These interventions were specifically designed for HCPs working with patients with cancer at end of life [26]. Most recently, face-to-face [27] and eLearning [28] interventions to promote family-centred cancer care at end of life for health and social care professionals have been developed and evaluated. These include a designated component regarding sharing information about the illness with dependent children [27, 28]. However, there remains an absence of training targeting HCPs’ role in the facilitation of family-centred conversations with all patients who have a serious illness (rather than exclusively focusing on cancer or end of life care).

To address this specific gap in HCPs’ knowledge, confidence and skills about initiating family-centred conversations across all medical specialties and professional roles, an intervention for HCPs was developed [10] using the Person-based Approach [29]. In summary, this involved a series of focus groups with (1) patients and partners with lived-experience of serious illness (2) adults with lived-childhood experience of a significant adult’s (e.g. parent, grandparent) serious illness (3) HCPs working with adult patients, to identify the content, structure and format of the intervention [10]. The intervention was a 5-minute animation (https://youtu.be/0esY5bsLM84) providing HCPs with a rationale for initiating family-centred conversations with adult patients. The intervention also included evidence about the benefits of talking with children about an adult’s serious illness and provided four practical steps for HCPs to follow when integrating this into their clinical practice. These steps are: (1) Identify the children who matter to the patient (2) Reassure patients that children need an explanation (3) Signpost families to practical guidance (weblinks included) (4) Check in with your patients [10]. Consistent with the Person-based Approach, there is a necessity to evaluate the acceptability, usefulness and effectiveness of an intervention [29] to better understand its potential impact on clinical practice.

This study aimed to evaluate the acceptability, usefulness and effectiveness of the intervention [10] (the animation) for HCPs working with adult patients who have a serious health condition and have significant relationships with children (under 18 years of age). The objectives were to:

1. evaluate HCPs’ self-reported knowledge, skills and confidence in facilitating family-centred conversations pre and post intervention.
2. explore the perceived acceptability and usefulness of the intervention for HCPs.
3. explore perceptions of how the intervention impacted healthcare clinical practice.

## Methods

### Patient and public involvement

A Patient and Public Involvement (PPI) group was convened at study inception (May 2020) comprising five individuals (4 female, 1 male) with lived-experience of their own, or a partner’s serious illness including leukaemia, cancer and inflammatory disease. One member of the group also had lived-childhood experience of parental serious illness. The PPI group had significant relationships with children ranging in age from 3 to 18 years at the time of the study. The project team included a PPI co-investigator who is a named co-author (JA). The PPI group members provided feedback on the grant application (including study design) and contributed to the planning and development stages of the intervention, as well as interpretation of results.

### Study design and setting

Consistent with the Person-based Approach [29] this study employed a multimethod evaluation of the intervention. Individuals were invited to complete an online quantitative survey immediately before and after accessing the intervention. Semi-structured qualitative interviews were then conducted online at least three months after individuals had engaged with the intervention to explore their usage, engagement, acceptability and effectiveness of the intervention for their clinical practice.

### Population

HCPs with experience of working with adult patients (age 18 years and older) who have a serious illness in a UK NHS setting within the previous three years. Recent NHS experience was chosen as an inclusion criterion to ensure participants had contemporary experience of clinical practice in the UK.

### Sampling

Volunteer sampling techniques were used to aid accrual to the study. Efforts were made to ensure recruitment of HCPs from different professional backgrounds through targeted approaches to professional organisations and leaders from different disciplines via email and tagging on social media. Participants who completed the survey could then volunteer to be contacted to take part in a qualitative interview.

### Recruitment

Adverts were distributed using social media platforms (including X) and through the research team’s professional networks. Individuals expressed interest in the study by responding to the recruitment advert which provided a link to the online participant information sheet and consent form hosted on JISC survey software. Following completion of the consent form, participants were automatically presented with the pre survey questions. The intervention was then embedded within the online JISC survey platform to enable participants to watch it before being automatically directed to the post survey questions. The page confirming successful submission of the post survey included an invitation to be contacted about a follow-up interview. Participants indicating their interest in this part of the study were then directed through to a separate survey site to leave their contact details. This step ensured that their survey responses were not linked to their email address. Three months after completion of the post survey, the research team (ER, LJD) sent an email (including the participant information sheet) inviting participants who had expressed an interest to take part in a one-off interview.

### Data collection

Following a systematic search of the literature by two authors (LJD, ER), no evaluation tools were identified that were specific to assess the aim and objectives of the intervention. In discussion with the clinical members of the research team, draft questions were developed and refined. All members emphasised the importance of brevity to avoid study fatigue and drop-out, especially amongst busy HCPs. The single-item questions developed related to a learning objective derived from the aims of the study.

A total of 255 participants completed the pre and post survey between April and July 2023. No data was recorded unless the final ‘submit’ button was clicked, meaning that drop-out during the surveys was not captured.

### Pre survey

Participants completed five questions about their current knowledge, self-rated skills and confidence about family-centred conversations on a 5-point Likert scale from 1 (strongly disagree, never) to 5 (strongly agree, always). ‘*Prefer not to say*’ was also included for each item.

### Post survey

The post survey comprised of the same five survey questions in the pre survey, but were re-phrased to ask about intended future practice. Three additional questions were asked about the perceived relevance and helpfulness of the intervention on a 5-point Likert scale from 1 (strongly disagree) to 5 (strongly agree). Finally, participants were asked to state their professional role prior to submitting their responses.

### Qualitative semi-structured interviews

Twenty-six participants indicated their willingness to be contacted regarding a follow-up interview. Invitations to arrange a convenient date and time were sent by the research team 12-weeks after the participants had viewed the animation and completed the pre and post surveys. There were nine non-responders, and one individual who later declined to be interviewed due to time commitments. Sixteen semi-structured interviews were conducted between August 2023 and November 2023. A topic guide (Supplementary material) was developed (by ER, LJD, SR, RH and JA) informed by the aims and objectives of the study, as well as the prior experience of the research team. Interviews were completed when no further categories were identified. Interviews took place online using MS Teams and were audio recorded. Interviews were completed by ER and LJD, neither of whom had prior relationships with the participants. Interviews lasted between 20 and 50 min (mean = 33.8 min). To demonstrate reflexivity, ER and LJD documented detailed reflections after each interview, which were discussed at regular team meetings throughout the data collection period. The topic guide was updated to include specific prompts about patients’ responses to participants initiating family-centred conversations and further exploration of how participants thought the intervention could be enhanced or supported by additional materials.

### Data analysis

#### Quantitative data

Survey responses were analysed by AS using R (version 4.4.2). Responses to each of the five survey items were reported on a five-point Likert scale (either strongly disagree to strongly agree, or never to always). The responses were converted to numeric values, 1 to 5, with higher scores indicating stronger agreement with the survey statement. Responses of ‘Prefer not to say’ were excluded from the analysis. For each item, mean scores and standard deviation (SD) were calculated for pre and post intervention. Difference scores (post minus pre) were computed, and normality was assessed using the Shapiro–Wilk test. As the assumption of normality was not met, non-parametric Wilcoxon signed-rank tests were conducted to evaluate differences between pre and post scores. Statistical significance was set at *p* < 0.05.

#### Qualitative data

Interview recordings were transcribed verbatim by the research team (ER and LJD). Reflexive thematic analysis guided the analysis of the interviews [27, 28]. ER and LJD read and re-read the transcripts to gain a sense of each discussion. Deployed as an inductive method, codes were developed by marking similar phrases or words as well as an analytical mind mapping process to explore patterns and relationships in the data and to identify themes [30, 31]. Final themes were verified and refined through critical dialogue with JA and SR.

### Ethics

Participants were provided with written information about the study and all provided written informed consent to participate and for their data to be published. This study was reviewed and approved by University of Oxford Medical Sciences Interdivisional Research Ethics Committee (MS IDREC) with approval number R74959/RE001 dated 10th June 2022.

## Results

### Pre and post survey

**Fig. 1:**
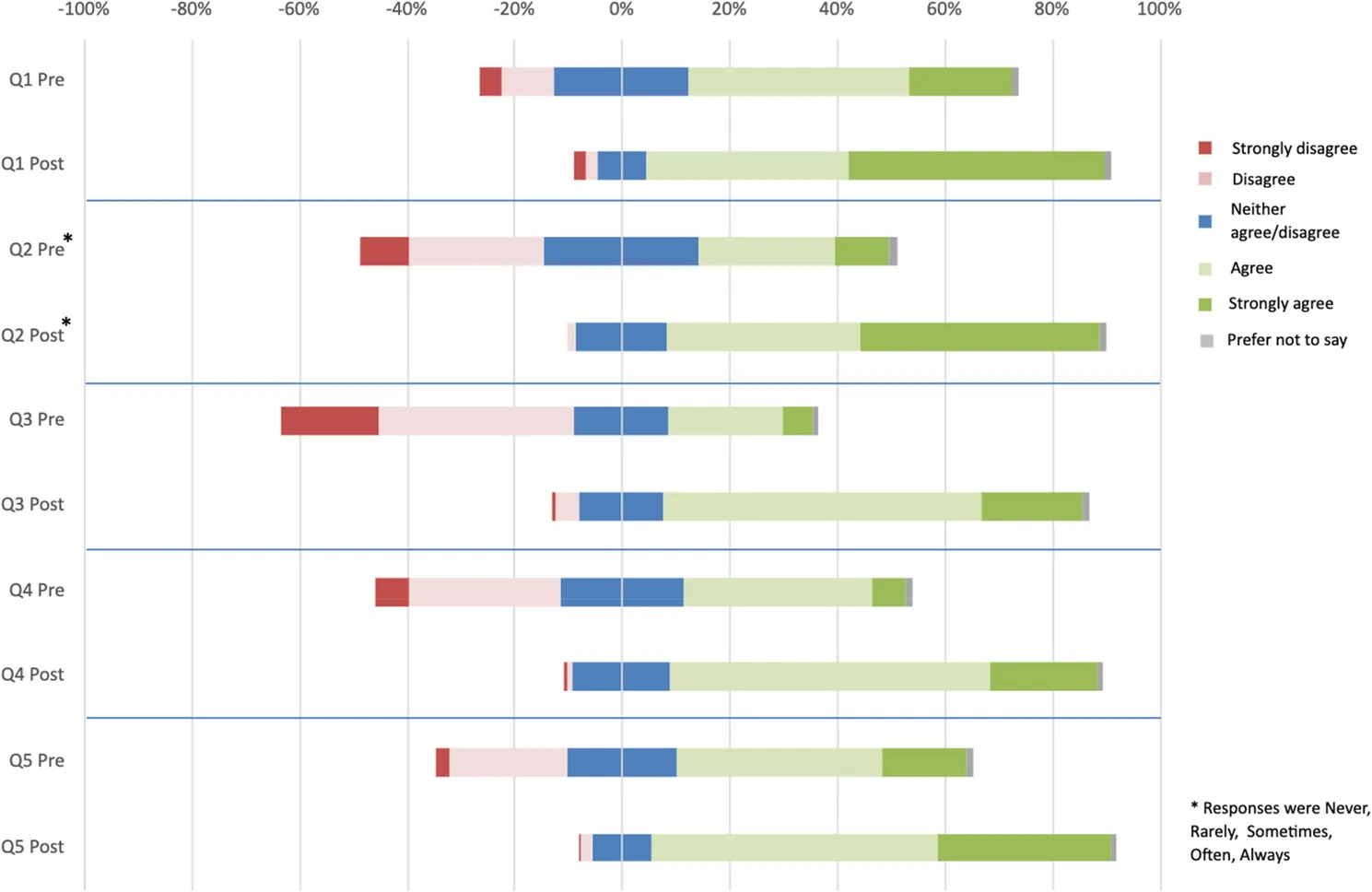
Distribution of survey responses pre and post intervention (*n* = 255)

A total of 255 HCPs completed the pre and post survey questions, including 114 doctors, 80 nurses and 30 allied health professionals (Table 1). Overall, the intervention yielded positive shifts in participants’ attitudes and intentions regarding family-centred conversations. A 100% stacked column chart was used to compare the distribution of responses to each survey question pre and post intervention, allowing visualisation of changes in response patterns (Fig. 1). There was an increase in the percentage of participants who ‘agreed’ or ‘strongly agreed’ that discussing children with adults with a serious illness was their responsibility (from 60.0% pre to 85.5% post intervention; question 1). There was also an increase in HCPs intending to ask adult patients about communicating with their children about the illness (from 35.3% pre to 80.4% post intervention; question 2). Participants also reported greater familiarity with the research on this topic (26.7% agreed or strongly agreed pre intervention, to 78% post intervention; question 3). There were also increases in participants’ knowledge and skills (41.1% agreed or strongly agreed pre and 79.6% post; question 4) and confidence to raise ‘telling children about the illness’ with patients (54.1% agreed or strongly agreed pre and 85.5% post; question 5).

**Table 1:** Outline characteristics of the 255 participants completing the survey

Healthcare Professional Role	*N* (%)
Doctor/Consultant	114 (44.7)
Nurse	80 (31.4)
Allied Healthcare Professional	30 (11.8)
Other*	13 (5.1)
Social Worker	11 (4.3)
Prefer not to say	5 (2.0)
Manager	2 (0.8)

*Other role reported to be Advanced Clinical Practitioner; Health Care Assistant; Spiritual Care chaplain; Pharmacist; Physiotherapist; Social Support Worker; Clinical Educator; Counsellor; Genetic Counsellor; Psychologist; Did not state (*n* = 3)

The mean survey question scores pre and post intervention are summarised in Table 2. Wilcoxon signed-rank test showed a significant increase from pre to post intervention for all five survey questions (*p* < 0.001).

**Table 2:** Mean item scores for the five survey questions asked pre and post intervention (*n* = 255)

	Survey Question	Mean score (SD)	Change in mean score	*p*-value^a^
Q1 Pre	When talking to my adult patients, it is my responsibility to raise the idea of how they might discuss their illness with any children in their life	3.62 (1.03)	0.66	< 0.001
Q1 Post	Having watched the animation, I think it is my responsibility to raise the idea of how my adult patients might discuss their illness with any children in their life	4.28 (0.89)		
Q2 Pre	I ask my adult patients “Have you thought about what you will tell the children in your life about your illness?”	3.02 (1.14)	1.22	< 0.001
Q2 Post	Having watched the animation, I now intend to ask my adult patients “Have you thought about what you will tell the children in your life about your illness?	4.25 (0.79)		
Q3 Pre	I am familiar with the research and evidence regarding talking to children about an adult’s illness	2.59 (1.17)	1.33	< 0.001
Q3 Post	Having watched the animation, I am familiar with the research and evidence regarding talking to children about an adult’s illness	3.92 (0.77)		
Q4 Pre	I have the knowledge and skills to help my adult patients think about ‘telling children about the illness’	3.07 (1.07)	0.91	< 0.001
Q4 Post	Having watched the animation, I have the knowledge and skills to help my adult patients think about ‘telling children about the illness’	3.98 (0.68)		
Q5 Pre	I am confident to raise ‘telling children about the illness’ with my adult patients	3.44 (1.07)	0.73	< 0.001
Q5 Post	Having watched the animation, I am confident to raise ‘telling children about the illness’ with my adult patients	4.16 (0.74)		

*SD* standard deviation

^a^Wilcoxon signed ranks test (non-parametric)

Feedback post intervention also revealed very positive attitudes about the intervention for clinical practice and 87.8% of participants recommending it to their colleagues (Table 3).

**Table 3:** Participants’ responses to the post survey questions six, seven and eight (*n* = 255)

Survey question and response options	% of participants
Q6: This animation is relevant to my clinical practice	
Strongly disagree	0.4
Disagree	1.2
Neither disagree or agree	7.4
Agree	43.3
Strongly agree	46.9
Prefer not to say	0.8
Q7: This animation will help my clinical practice	
Strongly disagree	0.4
Disagree	1.2
Neither disagree or agree	9.4
Agree	53.1
Strongly agree	35.1
Prefer not to say	0.8
Q8: I will recommend this animation to a colleague	
Strongly disagree	1.2
Disagree	1.6
Neither disagree or agree	9.0
Agree	44.9
Strongly agree	42.9
Prefer not to say	0.4

### Qualitative interviews

The sixteen participants were reflective of professionals working across nursing (*n* = 5), medical (*n* = 9) and allied health professional (*n* = 2) roles. Sample characteristics are reported in Table 4.

**Table 4:** Characteristics of the participants recruited to the qualitative interviews (*n*=16)

Healthcare Professional Role	*n*
Advanced Clinical Practitioner in Urgent Community Response	1
Anaesthetic Registrar	1
Clinical Psychologist in progressive neurological disease	1
Consultant in Gerontology	1
Consultant in the Neurosciences Intensive Care Unit	1
Consultant in Palliative Medicine	2
Consultant in Respiratory Medicine	1
General Practitioner	2
Oncology Clinical Nurse Specialist	2
Senior Sister in Critical Care	1
Social Support Worker in Critical Care	1
Strategic Lead Cancer Nurse	1
Surgeon	1

Findings are discussed under three themes: (1) HCPs’ perceptions of the intervention (2) Perceived impact of the intervention on clinical practice (3) Opportunities and challenges for family-centred conversations in routine practice.

### Theme 1: HCPs’ perceptions of the intervention

In general, participants were positive about the intervention reporting that the “*sensitive tone”* was appropriate to the subject matter and intended audience. Participants felt this was achieved through the music, hand drawn style of the animation and voiceover which fostered emotional connection. Participants consistently described the storyline and voiceover as an accurate reflection of their professional experience, which facilitated their engagement and investment in the intervention. Furthermore, participants reported that the short duration (5 minutes) made the intervention “*accessible to busy HCPs”*,* “achievable”* and “*right for the attention span of people*”.

*“I think every professional should watch this animation. I genuinely think that. You could do that with little cost to your time and take away an awful lot. It’s just very well done. It represents real life*,* it represents [HCPs] as we are*,* it represents our patients …The take home message is both clear by nature of the language used and the animation… and by the nature of the content as well”* Participant 11.

Many HCPs commented favourably on the large quantity of information that had been concisely presented in *“bite-sized chunks.”* The *“practical steps”* outlined in the intervention were considered “*easy”* for HCPs to follow when talking to patients, providing tangible and implementable “*take-aways”*. This included the provision of new, useful language and tools about ways to initiate family-centred conversations with patients. Furthermore, a pre-school child’s perspective demonstrated that even very young children notice changes in the family and need to be told about the illness. The link to an online patient-facing resource (step 3 of the intervention) was also valued by participants, with several reflecting that this could easily be downloaded or shared by text message with a family. Professionals commented favourably on the content of this additional resource and had incorporated it into their own practice. For example, discussing who might support a parent when they talk to their child, anticipating common reactions from children of different ages, and acknowledging the impact of the conversation on the adult themselves.

*Even [though] it’s a conversation that I have had before*,* but something about it* [the animation] *provided a really clarifying set of steps*,* for something that might feel a bit big or overwhelming for some healthcare professionals. And*,* so*,* I thought it might feel quite containing for people to feel like they could come away from that and think ‘I can do that*,* I can have that conversion with my patients whatever profession you are in”* Participant 16.

Some participants offered ideas about how to enhance the intervention. These included the need to emphasise the importance of telling children about the serious illness “*as soon as possible*”. This was considered especially important to avoid children finding out from other sources such as social media or overhearing conversations. Adjustments for neurodiverse children or those with learning disabilities were also considered to be a valuable area that could be included in the intervention. On occasion, it was reflected that simulations, which are often used in medical training, could be beneficial for rapidly facilitating integration into clinical practice.

*“I think animations are really good cause they give you a bit more distance*,* but . the things that really stick with me are actually watching simulations of it actually happening in front of you. .real conversations would be good…. So things like that*,* [HCPs] are quite fast when they see it real*,* they’re like ‘oh yeah’”* Participant 2.

### Theme 2: perceived impact of the intervention on clinical practice

Participants consistently reported that the intervention had prompted them to reflect on their own clinical practice about family-centred conversations. Frequently this included a recognition that they had not previously perceived this to be part of their clinical role. Many participants highlighted that hearing patients’ and children’s voices was a powerful, “*emotive and poignant*” reminder of the importance of both family relationships and children themselves. Some participants acknowledged that it was “*easy to lose sight”* of the patient’s family network and not consider the wider impact of the illness beyond “*the patient in front of them.”* Furthermore, many participants reported that the inclusion of characters of differing ages had prompted them to think about asking older patients, who might be grandparents, about their relationships with children.

*“Actually*,* you can have someone in emergency surgery who comes in with an unknown diagnosis of cancer*,* they’re dying*,* they will probably die. I have to say it had never even occurred to me at that point to even discuss children… and actually in hindsight*,* maybe I should have said*,* well*,* how do you deal with the children*,* the grandchildren that need to deal with it?”* Participant 17.

*“it’s [the intervention] just so helpful because it’s such a gap. I mean*,* I was almost embarrassed given my professional situation [HCP and communication skills trainer] and my personal situation [partner died when children were school age]. I was thinking ‘Oh my goodness; you don’t know what you don’t see’. I’ve been [pause] there’s a little bit of arrogance … I know my patients has two children*,* but I haven’t thought to mention to them ‘How are you managing to talk to your children? What would be helpful for you’?”* Participant 10.

Participants reported being more confident about raising the conversation with patients in routine practice following engagement with the intervention. In particular, participants felt that their new knowledge (regarding the benefits of effective communication for children’s outcomes) equipped them to advise patients to tell children about the illness, despite the family’s “*instinct*” of wanting to protect children from worry or distress. Participants reflected that while families might not often initiate conversations about children in clinic, many do want support with this difficult task. It was apparent that HCPs were more “*proactive”* and consistent in asking patients about important relationships with children post intervention. Several professionals reported that the positive response of families to their enquiries about children had reinforced their confidence to initiate family-centred conversations.

*“[since watching the intervention] we’ve started including ‘Are there any children you might need to talk to?’ in our sort of spiel that we add on to talking to the families. Because then people often say ‘Yeah*,* I really wanna tell my kids that mum is dying*,* but I just don’t quite know how to do that’”* Participant 2.

### Theme 3: opportunities and challenges for family-centred conversations in routine practice

Participants’ enthusiasm for the intervention was evident with many offering suggestions about how it could be disseminated to promote sustainable implementation of family-centred conversations. This included beliefs that the intervention should be integrated as part of mandatory training to promote universal awareness amongst staff and provide an annual *“prompt”* as to its importance. Some participants stated they had planned to include the intervention as part of the induction programme for new staff joining their clinical team. Perceptions about how they would continue to promote family-centred conversations in the workplace included using opportunities to share the intervention at their team’s journal club, lunchtime meeting or Quality Improvement Meetings.

*“There is something about it being ongoing*,* it should be mandatory every year. We have to learn about information governance*,* I think we should do something about comms to patients….this needs to be mandatory*,* this needs to be for everybody*,* every healthcare professionals’ vocabulary*,* because you know … we all have a relationship with children*,* don’t we?”* Participant 7.

Many participants suggested that the intervention and wider principle of family-centred communication should be included in existing professional training structures. There were a range of views about the most appropriate time during training to introduce this topic. Some believed it should be part of pre-registration curriculums in relevant health-related degree programmes. Others indicated the substantial emotional challenge experienced by junior staff when talking to patients about sensitive subjects, such as breaking bad news. Consequently, participants suggested that the intervention would be better placed later in professional education e.g. postgraduate education or continuing professional development courses. There was also acknowledgement of the important role played by senior HCPs in *“modelling behaviour”* to more junior members of the team. Participants highlighted both the challenge of changing established professionals’ behaviour, but also the potential power of senior leaders to promote family-centred practice within teams. It was suggested that including questions about family-centred communication in national patient experience surveys could be a useful strategy to raise the profile and perceived importance of this within services.

*“We don’t actually on any of the national standard patient experience surveys ask ‘Were you supported in how to tell your family or children’ I don’t think we ask that question and I think it’s a question we should ask”* Participant 7.

Participants gave practical suggestions about opportunities to embed the key messages of the intervention into routine care and ensure it remained a priority for busy HCPs. Several proposed that utilising existing patient record management systems could be a useful way to systematically remind HCPs to ask patients about children. Ideas from participants included setting alerts and prompts on these electronic systems which could be triggered by entering a diagnostic code, or tailored to specific demographics. Adding specific questions to clinical templates or checklists was also anticipated to be a useful strategy to remind HCPs to think about wider family relationships. Others recommended that posters in outpatient consulting rooms would serve as a useful reminder to HCPs “*Have we had the conversation about how they talk to children?”.* Participants also reflected that patients could be encouraged to “*put children on the agenda”* with their clinical team through the use of posters or videos on the screens in waiting areas.

*“If you put in a diagnostic code at a first diagnosis*,* you could just have an alert*,* it can flash up things*,* and it could say like ‘Who else is in the household*,* who could be affected by this diagnosis?’. I suppose that would be an alert to us wouldn’t it? So it’s a bit like if you put in depression it flags up alcohol ‘Does the patient drink*,* do they take drugs?’ So if an alert was given to a healthcare professional*,* that could be quite helpful*,* a prompt”* Participant 1.

Despite overwhelming enthusiasm for the intervention and embedding family-centred conversation into routine clinical care, a few participants commented on the ongoing obstacles to implementation. These included persistent uncertainty about whether all families would welcome a conversation about children and a concern that this would be contrary to a patient or family’s wishes. Others expressed a concern that time constraints or the absence of private spaces for sensitive conversations in some clinical settings make initiating family-centred conversations more challenging.

*“So like A&E would be …. I think is quite an inappropriate area because it’s so busy. It’s such a shock for whoever’s come in*,* what’s happening*,* that actually trying to get just the basic information of what’s happening to the patient across is very difficult to do. It’s very noisy environment then to ask about children …. just think might seem a bit inappropriate at that point. Not that it is inappropriate*,* but I just think it’s not quite the environment”* Participant 19.

A few participants reflected on a perceived broader avoidance within healthcare of acknowledging the implications of an illness for the patient or their family. For example, participants described medical letters and communication between professionals which used euphemisms, such as “*best supportive care*”, rather than explicitly articulating the cessation of curative treatment or transition to palliation. Participants also reported that clinical review letters do not include consideration of how the illness was impacting on children in the patient’s family. This was perceived to indicate the sizeable challenge of successfully embedding family-centred thinking and associated conversations into routine care.

*“I have not had a letter from any specialist nurse saying*,* ‘ohh I met this person for their annual Parkinson’s review and I talked to them about how their children were managing with their disease progression’. I have not had a letter that said that in epilepsy*,* neurology*,* COPD respiratory*,* chronic pain. Nothing. I might have a letter that says ‘they live with their children’……So I think just this sort of intervention in terms of raising the awareness is phenomenal*,* fantastic actually”* Participant 10.

## Discussion

This paper reports the multimethod evaluation of an online intervention [10] for HCPs developed to enhance their knowledge, skills and confidence to initiate family-centred conversations when an adult patient has a serious illness. Participants reported a significant increase in their (i) familiarity with evidence for the benefits of communication with children, (ii) knowledge, (iii) skills, and (iv) confidence in initiating family-centred conversations post intervention. Qualitative interviews with participants indicated that the intervention had resonated with their experiences as an HCP and provided an emotive reminder of the impact of illness on patients’ wider network. Participants reported that the intervention had prompted them to proactively ask patients about children and believed it should be embedded within existing training structures.

Over 90% of participants reported that the intervention was relevant to their clinical practice, with 87% indicating that they would recommend it to a colleague. Many participants suggested family-centred communication should be included in professional courses, induction packages or mandatory training. Time pressures frequently constrain HCPs’ engagement with CPD activities and communication skills training [32]; the brevity of this intervention may therefore facilitate its scalable dissemination across clinical services.

After participating in the intervention, 80.4% of HCPs reported that they intended to ask their patients “Have you thought about what you will tell the children in your life about your illness?” compared to 35.3% prior to the intervention. Whilst these results are very encouraging, embedding family-centred conversations into routine clinical care requires consideration of the potential drivers and obstacles to success. Implementation of research and quality improvement initiatives for HCPs’ practice have utilised a wide range of different models and strategies [33–36]. Many studies report a multifaceted approach and acknowledge both the interaction between different elements or strategies [34] and the influential role of local context [37]. The intervention in this study focused on individual-level factors; these were identified through the extensive development phases [10] including the interrogation of the published literature and thematic analyses of multiple focus groups with key stakeholders. The outputs of these activities informed the development of guiding principles and a logic model to ensure key elements were directly addressed through the format, content and features of the intervention itself [10]. Each of these components is discussed in turn.

Despite evidence consistently indicating that families want support and advice from HCPs about talking to children [38], many HCPs express uncertainty about whether patients would want them to raise the subject of children [11]. The intervention presented narratives of patients and people with lived-childhood experience sharing their desire for guidance from HCPs. Many participants reflected that these emotive stories had prompted a recognition of the systemic impact of the illness on patients’ families and the positive contribution HCPs could make. This aligns with the broader principles of HCP education [39] and the influential and enduring power of hearing first-hand patient experience in medical training [40, 41].

Previous research suggests that HCPs can make assumptions about whether to initiate family-centred conversations based on patients’ age with this being less likely for older patients [11, 14, 38]. The number of children potentially affected by an adult’s illness increases with each successive generation, with over 50% of grandparents providing formal or informal childcare for grandchildren [42]. This underlines the importance of identifying *every* patient’s significant relationships with children. Older characters (depicted through their facial appearance, posture, clothing, and the self-identification of a grandparent in a patient voiceover) prompted participants to recognise the importance of asking patients of all ages, driving changes to their clinical practice.

Perceived knowledge, skills and confidence are pivotal to HCPs’ engagement with family-centred conversations [11, 15] and therefore a clear target for training interventions [43]. The intervention was successful in increasing participants’ self-ratings across these three areas. Participants reported that their enhanced knowledge about the benefits of communication had directly impacted their “*comfort*” to advise patients to tell children about the illness. HCPs’ experiences of patients’ receptiveness to discussing children appeared to reinforce the routine integration of family-centred conversations into their clinical practice. Formal communication templates may facilitate HCPs’ confidence to begin initiating sensitive conversations with seriously ill patients [44, 45]; evidence indicates that after experiencing a positive response from patients, HCPs are more likely to make this part of their routine behaviour [44]. However, other studies have shown that an increase in knowledge, skills and confidence does not always correspond to an increase in support for families affected by serious illness [46].

While this intervention [10] was shown to address individual-level factors, ensuring the integration of family-centred conversations into routine care also requires strategies at the team, organisational, and system levels [35, 36]. Healthcare innovation requires organisational support, both from senior management and leaders within clinical teams [36]. Comparison of family-centred communication between different hospitals in Norway revealed that despite high reported knowledge, skills and confidence of HCPs, successful implementation of communication was related to leadership and co-worker support or supervision [46, 47]. HCPs who feel less certain about their role in family-centred care may be concerned about how their colleagues might perceive their decision to prioritise communication with families over other tasks (especially a more clearly defined professional role such as the physical care of a patient) [12]. Evidence suggests that the engagement and commitment of professional leaders is important to introduce novel or unusual practice [48]. Champions and opinion leaders can also be pivotal in facilitating adoption of new behaviours and attitudes [49, 50]. Without workplace support, including explicit, agreed expectations about who will deliver family-centred care, it becomes reliant on the beliefs and attitudes of individual HCPs [12]. Consequently, it is important for all team members to hold a shared understanding and agreement about their role in the routine implementation of this care. Participants suggested the intervention could be included in induction programmes; this could be an efficient way to ensure family-centred communication was highlighted to all new starters.

Consistent with a multifaceted implementation approach, participants offered various suggestions to help embed family-centred conversations in practice, including the addition of automated prompts and record keeping of conversations with patients. ‘Reminders’ can play a valued role within organisational implementation strategies [34]. Formal documentation can “*bring more attention to the conversation among the medical team*” [44], with others suggesting templates to record patients’ dependent children would reduce HCPs’ avoidance of this sensitive area of care [12].

There may be value of recruiting patients and their families as ‘partners’ in initiating family-centred conversations through the use of posters in clinical areas aimed at both HCPs and patients. Empowering patients to remind HCPs about important aspects of care has been studied in relation to hand hygiene procedures [51]. Evidence regarding the effectiveness of patient-facing videos, posters and leaflets is inconsistent, with studies highlighting the power dynamic between patients and HCPs contributing to patients’ reluctance to speak up [52]. Posters may be effective in prompting or ‘nudging’ clinician behaviour; initiatives to enhance HCPs’ adherence to hand hygiene protocols show some evidence of the benefits of visual reminders [53] although multi-modal strategies are considered more robust and effective [33].

### Strengths and limitations

The well recognised time constraints for professional training and skill development among HCPs continue to present a significant challenge [10, 32]. Crucially, despite the intervention’s brevity, it was liked and well-received. Combined with its electronic format, this makes the intervention readily accessible for busy HCPs with limited time or budget for CPD. This could help to engage HCPs who do not perceive family-centred conversations to be part of their role [11] and therefore would be unlikely to engage with a longer training course or workshop. Furthermore, the evaluation included HCPs from a range of professional backgrounds, in contrast to previous studies which have included predominantly nurses and few physicians [18].

Although the evaluation yielded positive results, several important questions remain regarding the generalisability and robustness of these findings. The study population consisted of UK NHS professionals, which raises questions about the direct transferability of these results to other countries and healthcare systems. The number of participants who engaged but did not complete the evaluation is unknown, limiting insight into the views of non-completers. The recruitment strategy may have introduced a self-selection bias by attracting HCPs who already possessed an interest or awareness of family-centred communication. The absence of a control group limited the ability to attribute changes solely to the intervention, as other factors may have influenced participants’ responses. As outcomes were evaluated after a single viewing of the intervention, the potential impact of repeated exposure could not be assessed. A validated survey instrument was not used for the quantitative evaluation and relied on self-reported changes. Qualitative data was limited to self-reported practice rather than observed behavioural change or how HCPs may have influenced patients’ decisions regarding talking with children about illness.

## Conclusion

The intervention (a 5 min animation) was positively received by HCPs across professional disciplines. Survey responses indicated that immediately after engaging with the intervention, HCPs reported a significant increase in knowledge, skills and confidence to initiate family-centred conversations about serious illness. Longer term qualitative data provides provisional support for the intended aims of the intervention; future studies should evaluate the intervention using controlled or randomised designs and assess longer-term HCP behavioural and patient-related outcomes.

## Supplementary Information

Supplementary Material 1.

## Data Availability

Data are available on reasonable request from the corresponding author.

## Abbreviations

HCP: Healthcare professional
NHS: National Health Service
CPD: Continuing Professional Development
