# Supplementary material for: Evaluating an intervention for healthcare professionals to facilitate family-centred conversations when an adult patient has a serious illness
Source: BMC Med Educ. 2026 Jul 17;26:1624. doi: 10.1186/s12909-026-09825-0 (PMC13656571; doi:10.1186/s12909-026-09825-0)
Supplement: Supplementary file 1 [file 12909_2026_9825_MOESM1_ESM.docx]

**Supplementary material**

Topic Guide for HCP interviews

|  | Prompts |
| --- | --- |
| Feedback on the form, content and presentation of the intervention | Explore likes, dislikes, anything that was missing, confusing or unclear  Explore suggestions for potential improvement |
| Relevance to clinical practice | Previous knowledge and experience of family-centred conversations  Any changes to practice since intervention; any patient groups you now ask about children, don’t ask about children and why |
| Patient response to family-centred conversations | How have patients responded to initiation of any family-centred conversations?  Have you shared the guides with any families? |
| Dissemination of intervention and embedding family-centred conversations in clinical care | Have you discussed the intervention or family-centred conversations with any colleagues?  Have you shared the intervention with anyone else?  Ideas about how the intervention could be disseminated  Suggestions of other resources that would be useful for HCPs or families  Ideas about embedding family-centred conversations within clinical care; learning from other successful initiatives within healthcare |
